# Deprescribing or represcribing: not just a semantic dilemma

**DOI:** 10.1007/s41999-021-00583-4

**Published:** 2021-11-05

**Authors:** Martin Wehling, Mirko Petrovic

**Affiliations:** 1 Clinical Pharmacology Mannheim, Centre for Gerontopharmacology, University of Heidelberg, Heidelberg, Germany; 2 Section of Geriatrics, Department of Internal Medicine and Paediatrics, Ghent University, Ghent, Belgium

Older people consume medicines in large quantities and many initiatives aim at reducing this drug load often called polypharmacy in this population. Given the fact that polypharmacy often leads to inappropriate prescribing and drug-related problems, various instruments have been developed to identify potentially inappropriate medications (PIM), such as the well-known and frequently updated Beers list [1] or other negative lists (e.g., STOPP, Hong Kong-specific criteria [2, 3]). They indicate what an older person should NOT be prescribed or should be stopped if already prescribed. Consequently, such PIM lists should support the approach that became famous as “deprescribing” [4]. This term has gained such popularity that it appears as a fashionable catchword to mark one of the most important areas of geriatric medicine. The recent Athens EuGMS congress (October 11–13 2021) addressed deprescribing in several lectures [3, 4].

Deprescribing studies do not really show what is the ultimate goal of patient studies, a robust improvement of clinical end points that seems highly desirable, as outcomes are quite negatively associated with polypharmacy, including functional assessments and mortality [5, 6]. This does not come as a surprise, as randomized controlled trials (RCT) on PIM lists largely failed regarding clinical outcomes [7]. In the systematic review of listing tools to improve medication in older people, another approach was also investigated, namely listing approaches that do not only identify overtreatment by PIM, but also positively label drugs or actions on drugs that may have been omitted (potentially omitted drugs POM). The two main lists—FORTA and START/STOPP—showed clinical benefits in RCTs and therefore clearly showed that deprescribing by itself is not always sufficient to improve an older patient’s clinical status. To mark the main difference between these and PIM lists, a crucial property of FORTA and START/STOPP was used for differentiation: PIM lists may be applied without knowing the patients’ characteristics, medical needs and personal preferences, but the medication scheme is all you need for their application. These lists were given the acronym DOLA (drug-oriented listing approach), as opposed to those POM/PIM positive–negative lists that may only be meaningfully applied if all this information is available, named PILA (patient-initiated listing approach).

The only PILA that is a mere drug list is FORTA, whereas START/STOPP addressed both action points (short excerpts from guideline actions) and drugs.

Such lists thus do not only lead to deprescribing of PIM, but also address the treatable needs of a patient and thus lead to new prescriptions if POM are identified. In the main RCT on FORTA, the VALFORTA-trial [8], the numbers of PIM and POM were almost equal at about three per patient; thus, the total number of eight drugs per patient and therefore the status of polypharmacy did not change. What a disappointment—no reduction of total drug load, but patients experienced clinical improvements, and less side effects, better Barthel index and more. At the 2016 EuGMS meeting, one of the authors of this editorial (MW) therefore coined the term represcribing as being more appropriate to address medication problems in older patients; they do have chances to receive positively labelled drugs for certain diseases (the FORTA list contains about 40% of positive labels), and they have all ethical and legal rights to have such treatments prescribed. Even a computer algorithm could identify undertreatment of pain as the most prevalent POM problem [9]. Furthermore, undertreatment (POM) has been linked to increased mortality rather than overtreatment [10]; though this association study should be corroborated by a RCT in the future, undertreatment at least appears to be an equally important issue compared to overtreatment (PIM).

However, these POM/PIM recommendations have to be implemented and realized in practice to gain positive clinical impact. Even the START/STOPP criteria failed in two larger trials (SENATOR, OPERAM [11, 12]) as PIM/POM recommendations were ineffectively transformed into clinical practice: in the latter study only 0.93 STOPP and—even worse—only 0.22 START recommendations per patient were realized in the end. No wonder that even a preferable PILA did not work, given this insufficient impact on prescription, in particular on starting POM prescriptions.

The older and also these very recent findings on clinical impact of listing approaches underline that deprescribing is only half the story—we should really start thinking a more appropriate novel term to cover the entire story, namely represcribing—appropriate drugs in, inappropriate drugs out. Clearly, in a patient on 25 medications, detoxification (inappropriate drugs out) would presumably be prevalent, but in average older people seem to have as many inappropriate drugs as they are missing the appropriate ones.

To better cope with these two sides of the coin, represcribing might be considered as the new catchword, that is “desprescribing 2.0” if you wish. The new catchword should facilitate ambitions to scientifically and clinically address both the main aspects of drug treatment for the sake of older patients—over- and undertreatment.
